# 6-month follow-up of VIALE-C demonstrates improved and durable efficacy in patients with untreated AML ineligible for intensive chemotherapy

**DOI:** 10.1038/s41408-021-00555-8

**Published:** 2021-10-01

**Authors:** Andrew H. Wei, Panayiotis Panayiotidis, Pau Montesinos, Kamel Laribi, Vladimir Ivanov, Inho Kim, Jan Novak, Don A. Stevens, Walter Fiedler, Maria Pagoni, Julie Bergeron, Stephen B. Ting, Jing-Zhou Hou, Achilles Anagnostopoulos, Andrew McDonald, Vidhya Murthy, Takahiro Yamauchi, Jianxiang Wang, Brenda Chyla, Yan Sun, Qi Jiang, Wellington Mendes, John Hayslip, Courtney D. DiNardo

**Affiliations:** 1 The Alfred Hospital and Monash University, Melbourne, VIC Australia; 2 National and Kapodistrian University of Athens Medical School, Laiko General Hospital, Athens, Greece; 3 Hospital Universitario y Politécnico La Fe, Valencia, Spain; 4 CIBERONC, Instituto Carlos III, Madrid, Spain; 5 Centre Hospitalier–Le Mans, Le Mans, France; 6 Almazov National Medical Research Center, Saint Petersburg, Russia; 7 Seoul National University Hospital, Seoul, South Korea; 8 University Hospital Královské Vinohrady and Third Faculty of Medicine, Charles University, Prague, Czech Republic; 9 Norton Cancer Institute, Louisville, KY USA; 10 Hubertus Wald University Cancer Center, University Medical Center Hamburg-Eppendorf, Hamburg, Germany; 11 Evaggelismos General Hospital, Athens, Greece; 12 CIUSSS-EMTL, Installation Maisonneuve-Rosemont, Montreal, QC Canada; 13 Eastern Health and Monash University, Melbourne, VIC Australia; 14 University of Pittsburgh Medical Center (UPMC) Cancer Center, Pittsburgh, PA USA; 15 George Papanicolaou General Hospital, Thessalonica, Greece; 16 Netcare Pretoria East Hospital, Moreletapark, Pretoria, South Africa; 17 Heartlands Hospital, Birmingham, UK; 18 University of Fukui Hospital, Fukui, Japan; 19 Chinese Academy of Medical Sciences, Tianjin, China; 20 AbbVie Inc, North Chicago, IL USA; 21 The University of Texas MD Anderson Cancer Center, Houston, TX USA

**Keywords:** Targeted therapies, Acute myeloid leukaemia

## Abstract

VIALE-C compared the safety and efficacy of venetoclax or placebo plus low-dose cytarabine (+LDAC) in patients with untreated AML ineligible for intensive chemotherapy. Overall, 211 patients were enrolled (*n* = 143, venetoclax; *n* = 68, placebo). At the primary analysis, the study did not meet its primary endpoint of a statistically significant improvement in overall survival (OS), however, ~60% of patients had been on study for ≤6-months. Here, we present an additional 6-months of follow-up of VIALE-C (median follow-up 17.5 months; range 0.1–23.5). Median OS was (venetoclax +LDAC vs. placebo +LDAC) 8.4 vs. 4.1 months (HR = 0.70, 95% CI 0.50,0.99; *P* = 0.040); a 30% reduction in the risk of death with venetoclax. Complete response (CR)/CR with incomplete hematologic recovery (CRi) rates were 48.3% vs. 13.2%. Transfusion independence rates (RBC) were 43% vs.19% and median event-free survival was 4.9 vs. 2.1 months (HR = 0.61; 95% CI 0.44,0.84; *P* = 0.002). These results represent improved efficacy over the primary analysis. Incidence of grade ≥3 adverse events were similar between study arms and overall safety profiles were comparable to the primary analysis. These data support venetoclax +LDAC as a frontline treatment option for patients with AML ineligible for intensive chemotherapy.

This trial was registered at www.clinicaltrials.gov as #NCT03069352.

## Introduction

The incidence of acute myeloid leukemia (AML) in the United States is estimated to be 4.3 per 100,000 people per year, with a death rate of 2.8 per 100,000 people annually [1]. AML is most common in older adults (aged >60 years) [1, 2], and elderly patients (aged ≥75 years), or younger patients with comorbidities, are often ineligible to receive standard intensive chemotherapy due to high rates of toxicity and early mortality. Consequently, approximately 60% of adults aged ≥75 years or those aged <75 years with comorbidities receive only supportive care following diagnosis [3, 4]. For those receiving treatment, less intensive options include hypomethylating agents (HMAs; such as azacitidine and decitabine) or low-dose cytarabine (LDAC), although composite remissions occur in <30% of patients, with median overall survival (OS) times of approximately 4 months with LDAC and 7–10 months with HMAs [5–8]. Thus, there is a clear need for alternative therapeutic approaches for these subsets of patients.

Venetoclax is an orally bioavailable small molecule that selectively and potently inhibits B-cell lymphoma 2 (BCL-2) activity [9]. BCL-2 overexpression has been associated with resistance to chemotherapy and poorer outcomes in patients with AML [10]. Combination therapy of venetoclax plus LDAC in older adults (aged ≥60 years) ineligible for intensive chemotherapy has shown a 54% complete response (CR)/CR with incomplete hematologic recovery (CRi) rate and a median OS for all patients of 10.1 months [11].

Recently, the phase 3 VIALE-C study (NCT03069352) (ref [12]) assessed the safety and efficacy of venetoclax plus LDAC compared with placebo plus LDAC in treatment-naive patients with AML who were considered ineligible for intensive chemotherapy. Although this study did not meet its primary endpoint of a statistically significant improvement in OS (hazard ratio [HR] 0.75, 95% confidence interval [CI] 0.52, 1.1, *P* = 0.11), the primary analysis did demonstrate prolonged median OS in patients who received venetoclax versus those who received placebo (7.2 vs. 4.1 months, respectively), as well as a 25% reduction in the risk of death [12]. At the time of primary analysis, 82/143 (57%) patients in the venetoclax arm and 48/68 (62%) patients in the placebo arm had been on study for ≤6 months. Here, we report safety and efficacy data from *post hoc* analyses performed following an additional 6-months of follow-up of the VIALE-C study.

## Materials and methods

### Study design and patients

This randomized, double-blind, placebo-controlled, multicenter phase 3 study (NCT03069352) evaluated the efficacy and safety of venetoclax plus LDAC combination therapy (subsequently referred to as simply venetoclax) compared with placebo plus LDAC (subsequently referred to as simply placebo) in patients with AML who had not received prior AML treatment and were ineligible for intensive chemotherapy. The study design for VIALE-C has been reported previously [12].

The primary objective of the study was to assess if venetoclax treatment led to an improvement in OS compared with placebo. Secondary endpoints included response rates: CR, CR/CRi, and CR/CR with partial hematologic recovery (CRh); the proportion of patients achieving CR/CRi, or CR/CRh by the beginning of cycle 2; transfusion independence rates; event-free survival (EFS); and minimal residual disease (MRD).

Details on patient eligibility criteria have been published previously [12]. In brief, the study enrolled patients with histologically confirmed AML, according to World Health Organization criteria [13], who were ineligible for intensive induction chemotherapy. Patients were either ≥75 years of age or were younger (18–74 years) and fulfilled at least one criterion associated with lack of fitness for intensive induction chemotherapy. Patients who received prior treatment for AML (except for hydroxyurea either prior to or during the first cycle of treatment) were excluded as were those previously treated with cytarabine for any indication.

Local ethics committee approval was obtained, and all patients provided written informed consent. The study was conducted in accordance with the International Conference on Harmonization, Good Clinical Practice guidelines, and the Declaration of Helsinki.

### Treatment

Eligible patients were randomized 2:1 to receive either venetoclax or placebo. To mitigate the risk of tumor lysis syndrome (TLS), escalating doses of venetoclax were administered in a hospital setting during a 4-day ramp-up period at the beginning of cycle 1. Patients received 100 mg venetoclax orally on day 1, 200 mg on day 2, 400 mg on day 3, and 600 mg daily on days 4–28 of cycle 1 and daily in all subsequent 28-day cycles. Patients remained in the hospital until 24 hours after receiving the maximum 600 mg dose of venetoclax or placebo. Additionally, all enrolled patients received TLS prophylaxis during the ramp-up period including hospitalization, administration of oral and intravenous hydration, treatment with a uric acid–reducing agent, laboratory assessments, and close monitoring. Patients randomized to the placebo arm were administered placebo in the same manner as venetoclax. All patients received LDAC (20 mg/m^2^ subcutaneously) on days 1–10 of each 28-day cycle. Patients continued to receive study treatment until investigator-assessed disease progression, unacceptable toxicity, withdrawal of consent, or the meeting of other pre-determined treatment discontinuation criteria as published previously [12].

### Study assessments

*Safety*. Safety assessments were conducted as reported previously [11]. Briefly, adverse events (AEs) and serious AEs (SAEs) emerging between day 1 of venetoclax or placebo and LDAC treatment and 30 days after the last dose of study treatment were recorded and graded according to the National Cancer Institute Common Terminology Criteria for Adverse Events v4.03. Laboratory-confirmed TLS was defined as reported previously [14].

*Efficacy*. Response assessments were performed on bone marrow biopsies collected at screening, at the end of cycles 1 and 4, and every 3 cycles thereafter until disease progression, or until two successive samples indicated CR or CRi [15]. Response rates were assessed according to modified International Working Group criteria for AML [16]. Criteria for evaluating primary and secondary outcome measures have been defined in detail previously [12]. Briefly, OS was defined as the number of days from study randomization to death, and EFS was defined as the number of days from study randomization to disease progression, relapse from CR or CRi, treatment failure, or death from any cause. Treatment failure was defined as failure to achieve a morphologic leukemia-free state (MLFS) or higher response (CR, CRi, or PR). Post-baseline transfusion independence, of either red blood cells (RBCs) or platelets, was defined as ≥56 consecutive days without transfusions, occurring between study drug initiation and 30 days after study drug completion. An MRD response was defined as having <10^−3^ residual blasts per leukocytes in the bone marrow as per European LeukemiaNet recommendations [17]. After the achievement of CR/CRi, further marrow assessments for MRD were not mandated, per protocol. After discontinuation of study treatment, patients will be assessed for OS, disease progression, and post-therapy disease status every two months until the end of the study, or for two years after enrollment of the last patient in the study for those patients who achieved a CR, CRi, PR, or MLFS.

### Statistical analyses

The data cut-off for the 6-month follow-up was August 15, 2019. All patients who received at least one dose of study drug (venetoclax or placebo) were included in safety analyses (*N* = 210), whereas all patients who were randomized were included in efficacy analyses (*N* = 211). A sample size of 210 patients was pre-planned to detect a statistically significant reduction in mortality of 45.5% in patients receiving venetoclax compared with placebo, with 90% power at an alpha level of 0.05. Information on endpoint analyses have been reported previously [12].

### Data sharing statement

AbbVie is committed to responsible data sharing regarding the clinical trials we sponsor. This includes access to anonymized, individual, and trial-level data (analysis data sets), as well as other information (e.g., protocols and Clinical Study Reports), as long as the trials are not part of an ongoing or planned regulatory submission. This includes requests for clinical trial data for unlicensed products and indications.

This clinical trial data can be requested by any qualified researchers who engage in rigorous, independent scientific research, and will be provided following review and approval of a research proposal and Statistical Analysis Plan (SAP) and execution of a Data Sharing Agreement (DSA). Data requests can be submitted at any time and the data will be accessible for 12 months, with possible extensions considered. For more information on the process, or to submit a request, visit the following link: https://www.abbvie.com/our-science/clinical-trials/clinical-trials-data-and-information-sharing/data-and-information-sharing-with-qualified-researchers.html.

## Results

### Patient demographics and clinical characteristics

Of the 211 patients enrolled, 143 were randomized to the venetoclax arm and 68 to the placebo arm. One patient randomized to the venetoclax arm did not receive treatment. Key demographics and clinical baseline characteristics for patients at the pre-planned primary analysis have been reported previously [12]. Briefly, patients had a median age of 76 years, were predominantly male (55.5%), 38.4% had secondary AML, 19.9% had prior HMA treatment, and 32.8% had poor cytogenetic risk. Baseline patient characteristics were relatively well balanced between the study arms, although a greater frequency of patients in the venetoclax arm had secondary AML (40.6% vs. 33.8%), poor cytogenetic risk (34.1% vs. 30.3%), and history of myelodysplastic syndrome (32.9% vs. 25.0%) compared with the placebo arm.

### Duration on study

As of the 6-month follow-up data cut-off date, 103 patients (72.0%) in the venetoclax arm and 56 patients (82.4%) in the placebo arm had discontinued the study. The median time on study was 17.5 months (range 0.1–23.5) vs. 17.7 months (range 0.2–20.8) for patients in the venetoclax and placebo arms, respectively. The median duration of venetoclax treatment was 4.1 months (range <0.1–23.5) compared with 1.7 months (range 0.1–20.2) for placebo treatment. Patients in the venetoclax arm received a median of 4.0 treatment cycles compared with those in the placebo arm who received a median of 2.0 treatment cycles. The median duration for LDAC treatment was 3.5 months (range <0.1–23.4) in the venetoclax arm compared with 1.3 months (range <0.1–19.9) in the placebo arm.

Of the 76 patients in the venetoclax arm who achieved a best response of CR, CRi, or MLFS, 28 (36.8%) had a venetoclax dose interruption due to blood count recovery. The same number and proportion had an LDAC dose interruption, also due to blood count recovery. A total of 9 patients (11.8%) had one venetoclax dose interruption, 10 patients (13.2%) had two, and 9 patients (11.8%) had more than two venetoclax dose interruptions due to blood count recovery. Likewise, a total of 9 (11.8%) patients had one LDAC dose interruption, 4 (5.3%) had two dose interruptions, and 15 (19.7%) patients had more than two LDAC dose interruptions due to blood count recovery. No patients in the placebo arm who achieved the best response of CR, CRi, or MLFS (*n* = 11) had placebo or LDAC dose interruptions due to blood count recovery.

Overall, 180 patients (85.3%) discontinued either venetoclax (*N* = 117 [81.8%]) or placebo (*N* = 63 [92.6%]) treatment. Reasons for study drug discontinuation are described in Supplementary Information Table S1. The most common reasons for treatment discontinuation in the placebo arm were treatment failure and disease progression. Within the venetoclax arm morphologic relapse was the most common reason.

### Safety profile

Overall, the safety profiles observed in the venetoclax and placebo arms were comparable to those reported in the primary analysis [12]. AEs present in ≥20% of patients are summarized in Table 1. Similar frequencies of AEs were reported in both study arms with 141 patients (99%) in the venetoclax arm and 67 patients (99%) in the placebo arm reporting at least one AE. The most frequently reported all-grade AEs were neutropenia, thrombocytopenia, and nausea which all occurred at a higher frequency in patients in the venetoclax arm (49%, 46%, and 43%, respectively) compared with patients in the placebo arm (18%, 40%, and 31%, respectively). Grade ≥3 AEs were also comparable between study arms with 138 patients (97%) in the venetoclax arm and 65 patients (96%) in the placebo arm experiencing at least one grade ≥3 AE. The most frequent grade ≥3 AEs were neutropenia, thrombocytopenia, and febrile neutropenia which all occurred at a higher frequency in the venetoclax arm (49%, 46%, and 32%, respectively) compared with patients in the placebo arm (18%, 38%, and 29%, respectively). However, SAEs related to those hematologic AEs (sepsis, pneumonia, etc.) were similar in both treatment arms.

**Table 1:** Summary of AEs by MedDRA SOC.

AE, *n* (%)	All-grade AEs, ≥ 20% of total patients		Grade ≥ 3 AEs, ≥ 20% of total patients		SAEs, ≥ 10% of total patients	
	VEN + LDAC (*n* = 142)	PBO + LDAC (*n* = 68)	VEN + LDAC (*n* = 142)	PBO + LDAC (*n* = 68)	VEN + LDAC (*n* = 142)	PBO + LDAC (*n* = 68)
Any	141 (99)	67 (99)	138 (97)	65 (96)	95 (67)	42 (62)
Hematologic	115 (81)	51 (75)	111 (78)	50 (74)	33 (23)	16 (24)
Neutropenia	69 (49)	12 (18)	69 (49)	12 (18)	4 (3)	0
Thrombocytopenia	65 (46)	27 (40)	65 (46)	26 (38)	7 (5)	2 (3)
Febrile neutropenia	46 (32)	20 (29)	46 (32)	20 (29)	24 (17)	12 (18)
Anemia	41 (29)	15 (22)	38 (27)	15 (22)	4 (3)	0
Gastrointestinal disorders	106 (75)	47 (69)	19 (13)	6 (9)	10 (7)	1 (1)
Nausea	61 (43)	21 (31)	2 (1)	0	0	0
Diarrhea	47 (33)	12 (18)	4 (3)	0	1 (1)	0
Vomiting	41 (29)	10 (15)	1 (1)	0	0	0
Constipation	29 (20)	22 (32)	1 (1)	0	0	0
Metabolism and nutrition disorders	87 (61)	40 (59)	40 (28)	22 (32)	5 (4)	0
Hypokalemia	44 (31)	17 (25)	17 (12)	11 (16)	0	0
Decreased appetite	31 (22)	13 (19)	2 (1)	0	1 (1)	0
Infections	92 (65)	41 (60)	61 (43)	34 (50)	53 (37)	25 (37)
Pneumonia	31 (22)	11 (16)	25 (18)	11 (16)	20 (14)	7 (10)

*AE* adverse event, *LDAC* low-dose cytarabine, *MedDRA SOC* medical dictionary for regulatory activities system organ class, *PBO* placebo, SAE, serious AE, *VEN* venetoclax.

The rate of death occurring within 30 days of study drug initiation was 13% in the venetoclax arm and 16% in the placebo arm. Fatal AEs occurred at similar frequencies in the venetoclax and placebo arms (23% vs. 21%, respectively). AEs categorized under the MedDRA system organ class preferred term of infections and infestations were linked to the highest incidence of death in both study arms (15% in venetoclax arm vs. 10% in placebo arm). Pneumonia (venetoclax vs. placebo; 5% vs. 0%), septic shock (4% vs. 4%), and sepsis (3% vs. 1%) were the most common infections and infestations leading to death.

### Efficacy

**Fig. 1:**
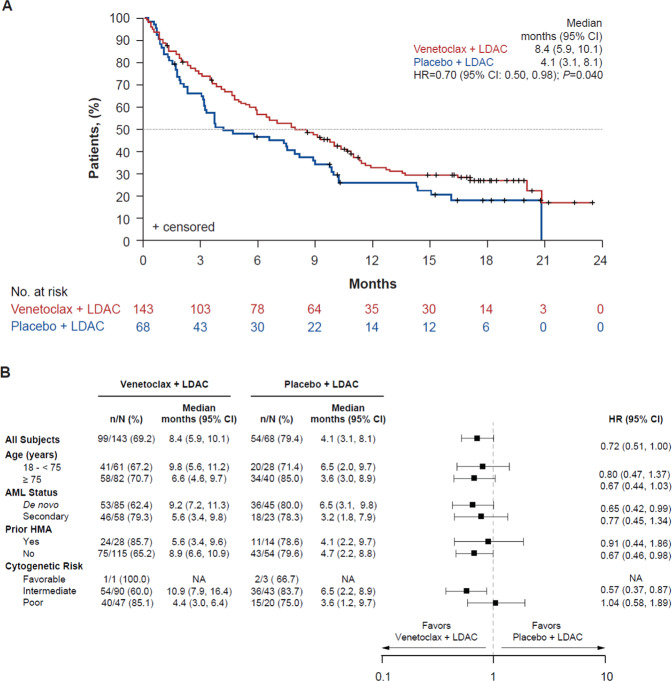
Overall Survival. **A** OS at 6-month follow-up. Kaplan–Meier plot showing the OS rate of all patients over time, separated by treatment arm; the number of patients at risk for each time point is shown below the graph. Tick marks indicate censored data. Republished with permission of Elsevier Science & Technology Journals, from Venetoclax plus LDAC for newly diagnosed AML ineligible for intensive chemotherapy: a phase 3 randomized placebo-controlled trial, Wei et al., volume 135, issue 24, copyright 2021; permission conveyed through Copyright Clearance Center, Inc. **B** Subgroup analysis of investigator-assessed OS. HR is from the unstratified Cox proportional-hazards model. Data included are subjected to a cut-off date of August 15, 2019. Median (95% CI) and HR (95% CI) are calculated only for subgroups with available data. AML acute myeloid leukemia, CI confidence interval, HMA hypomethylating agent, HR hazard ratio, LDAC low-dose cytarabine, NA not assessed, OS overall survival.

At the completion of the 6-month follow-up, median OS was longer in patients in the venetoclax arm (8.4 months, 95% CI 5.9, 10.1) compared with those in the placebo arm (4.1 months, 95% CI 3.1, 8.1) (HR 0.70, 95% CI 0.50, 0.98, *P* = 0.040) (Fig. 1A). The risk of death was reduced by 30% in patients in the venetoclax arm compared with those in the placebo arm. A *post hoc* stepwise multivariate Cox regression test was performed on the 6-month follow-up OS data to identify the influence of pre-treatment demographics and baseline disease characteristics associated with OS. A total of 5 covariates (treatment arm, age, AML status, Eastern Cooperative Oncology Group performance status score, and cytogenetic risk) were shown to be correlated with OS (Table 2). With a covariate-adjusted HR of 0.65 (95% CI 0.46, 0.91, *P* = 0.012), this sensitivity analysis demonstrated a beneficial treatment effect for venetoclax compared with placebo.

**Table 2:** Multivariate Cox regression analysis of OS in 6-month follow-up cohort^a^.

Covariate	Adjusted HR (95% CI)	*P* value
Treatment arm (venetoclax vs. placebo)	0.65 (0.46, 0.91)	0.012
Age group (<75 vs. ≥75 years)	0.59 (0.40, 0.86)	0.006
AML status (de novo vs. secondary)	0.61 (0.44, 0.86)	0.004
ECOG PS score (<2 vs. ≥2)	0.50 (0.35, 0.72)	<0.001
Cytogenetic risk (intermediate vs. poor)^b^	0.58 (0.41, 0.82)	0.002

^a^Baseline characteristics included in the stepwise variable selection: treatment arm, age, sex, AML status, bone marrow blast count, ECOG PS score, cytogenetic risk group, prior HMA use, geographic region, *FLT3* mutation status, *IDH1/2* mutation status, and *NMP1* mutation status. ^b^Favorable vs. poor cytogenetic risk is not presented in this table as only comparisons with *p*-value ≤0.05 were included.

*AML* acute myeloid leukemia, *CI* confidence interval, *ECOG PS* Eastern Cooperative Oncology Group performance status, *FLT3* FMS-related tyrosine kinase 3, *HMA* hypomethylating agent, *HR* hazard ratio, *IDH1/2* isocitrate dehydrogenase 1/2, *NMP1* nucleophosmin, *OS* overall survival.

Subgroup analysis of OS, including patients receiving prior HMA treatment versus those who did not, as well as patient cytogenetic risk (intermediate vs. poor), demonstrated that treatment with venetoclax improved OS compared with placebo across all subgroups analyzed (Fig. 1B). As of the 6-month follow-up, 153 deaths had been reported (99 patients [69.2%] in the venetoclax arm and 54 patients [79.4%] in the placebo arm).

Interestingly, rates of post-study treatment differed notably between the two arms with a lower frequency of patients in the venetoclax arm (29%) receiving any subsequent therapy compared to patients in the placebo arm (50%). A lower frequency of patients in the venetoclax arm (8%) received intensive chemotherapy after the study compared to patients in the placebo arm (22%).

Secondary efficacy analyses are summarized in Table 3. Remission rates for patients in the venetoclax arm were higher than in the placebo arm. The investigator-assessed CR rate at the 6-month follow-up was 28.0% in the venetoclax arm versus 7.4% in the placebo arm. Similarly, CR/CRi rates (venetoclax vs. placebo; 48.3% vs. 13.2%) and CR/CRh rates (48.3% vs. 14.7%) were also higher in patients in the venetoclax arm compared with patients in the placebo arm. Additionally, the median duration of response (DoR) was higher in patients in the venetoclax arm compared with patients in the placebo arm in terms of CR (17.1 vs. 8.3 months), CR/CRi (11.7 vs. 6.2 months), and CR/CRh (11.7 vs. 8.3 months, respectively).

**Table 3:** Summary of secondary endpoints.

Secondary endpoint	Venetoclax +LDAC (*n* = 143)	Placebo + LDAC (*n* = 68)	*P* value
***Remission rates, % (95% CI)***			
**CR**, % (95% CI)	28.0 (20.8, 36.1)	7.4 (2.4, 16.3)	<0.001
Median DoR, months	17.1	8.3	
**CR/CRi**, % (95% CI)	48.3 (39.8, 56.8)	13.2 (6.2, 23.6)	<0.001
Median time to first remission, months (range)^a^	1.1 (0.8, 16.3)	3.7 (0.9, 6.5)	
By initiation of cycle 2, % (95% CI)	34.3 (26.5, 42.7)	2.9 (0.4, 10.2)	
Median DoR, months	11.7	6.2	
**CR/CRh**, % (95% CI)	48.3 (39.8, 56.8)	14.7 (7.3, 25.4)	<0.001
Median time to first remission, months (range)^a^	1.0 (0.7, 16.3)	2.8 (0.9, 6.5)	
By initiation of cycle 2, % (95% CI)	30.8 (23.3, 39.0)	4.4 (0.9, 12.4)	
Median DoR, months	11.7	8.3	
***Post-baseline transfusion independence rates, % (95% CI)***			
RBC	43.4 (35.1, 51.9)	19.1 (10.6, 30.5)	<0.001
Platelet	49.0 (40.5, 57.4)	32.4 (21.5, 44.8)	0.024
Both	39.2 (31.1, 47.7)	17.6 (9.5, 28.8)	0.002
***Post-baseline transfusion independence rates by baseline transfusion status***^**b**^***, % (95% CI)***			
***RBC***			
Dependent at baseline	40.4 (30.9, 50.5)	16.7 (7.9, 29.3)	NP^c^
Independent at baseline	51.3 (34.8, 67.6)	28.6 (8.4, 58.1)	
***Platelet***			
Dependent at baseline	28.8 (17.1, 43.1)	12.5 (2.7, 32.4)	NP^c^
Independent at baseline	60.4 (49.6, 70.5)	43.2 (28.3, 59.0)	
***Both***			
Dependent at baseline	35.1 (26.3, 44.8)	14.3 (6.4, 26.2)	NP^c^
Independent at baseline	53.1 (34.7, 70.9)	33.3 (9.9, 65.1)	
**Median EFS, months (95% CI)**	4.9 (3.7, 6.4)	2.1 (1.5, 3.2)	0.002

^a^One patient in the venetoclax arm took 16.3 months to achieve their first response and this patient makes up the latter edge of the range described. All other patients in the venetoclax arm responded within 5 months. ^b^Baseline transfusion status: transfusion-dependent at baseline if RBC or platelet transfusion received within 8 weeks of the first dose of study drug; transfusion independent at baseline if RBC or platelet transfusion was not received within 8 weeks of the first dose of study drug. ^c^Per the statistical analysis plan (SAP), no statistical comparison was performed for conversion rates.

*CI* confidence interval, *CR* complete response, *CRh* complete response with partial hematologic recovery, *CRi* complete response with incomplete hematologic recovery, *DoR* duration of response, *EFS* event-free survival, *LDAC* low-dose cytarabine, *NP* not performed, *RBC* red blood cell.

At the 6-month follow-up of those patients who were RBC or platelet transfusion dependent at baseline (*n* = 111 venetoclax arm; *n* = 56 placebo arm), a higher frequency of patients in the venetoclax arm became RBC and platelet transfusion independent compared with patients in the placebo arm (35.1% vs. 14.3%, respectively) (Table 3). At baseline, 32 patients in the venetoclax arm and 12 patients in the placebo arm were independent of RBC or platelet transfusions and 17/32 (53.1%) versus 4/12 (33.3%) remained transfusion independent for ≥56 days post-baseline, respectively.

Higher rates of CR/CRi and improved EFS were observed in patients in the venetoclax arm compared with those in the placebo arm. Median EFS was 4.9 months in the venetoclax arm versus 2.1 months in the placebo arm (HR 0.61, 95% CI 0.44, 0.84, *P* = 0.002). Investigator-assessed efficacy analyses in several key subgroups are presented in Table 4.

**Table 4:** Analysis of investigator-assessed response rates by subgroup.

	Venetoclax +LDAC				Placebo + LDAC			
	N	CR, *n* (%)	CR/CRi, *n* (%)	CR/CRh, *n* (%)	N	CR, *n* (%)	CR/CRi, *n* (%)	CR/CRh, *n* (%)
***Age***								
18 to <75 years	61	17 (27.9)	28 (45.9)	27 (44.3)	28	2 (7.1)	4 (14.3)	4 (14.3)
≥75 years	82	23 (28.0)	41 (50.0)	42 (51.2)	40	3 (7.5)	5 (12.5)	6 (15.0)
***Cytogenetic risk***^**a**^								
Intermediate	90	29 (32.2)	51 (56.7)	50 (55.6)	43	4 (9.3)	7 (16.3)	8 (18.6)
Poor	47	8 (17.0)	13 (27.7)	15 (31.9)	20	1 (5.0)	2 (10.0)	2 (10.0)
***AML type***								
De novo	85	31 (36.5)	47 (55.3)	50 (58.8)	45	5 (11.1)	8 (17.8)	9 (20.0)
Secondary	58	9 (15.5)	22 (37.9)	19 (32.8)	23	0	1 (4.3)	1 (4.3)
***Prior HMA treatment***								
Yes	28	2 (7.1)	8 (28.6)	6 (21.4)	14	0	1 (7.1)	1 (7.1)
No	115	38 (33.0)	61 (53.0)	63 (54.8)	54	5 (9.3)	8 (14.8)	9 (16.7)

^a^Seven total patients (*n* = 5 venetoclax arm, *n* = 2 placebo arm) had missing cytogenetic risk profiles. Four total patients were deemed to have favorable cytogenetic risk (*n* = 1 venetoclax, *n* = 3 placebo) and their response data were not presented due to small sample size.

AML, acute myeloid leukemia, *CR* complete response, *CRh* CR with partial hematologic recovery, *CRi* CR with incomplete hematologic recovery, *HMA* hypomethylating agent, *LDAC* low-dose cytarabine.

Of particular interest, data from patients assessed for MRD at the 6-month follow-up are summarized in Table 5. Overall, 12 patients (8.4%) in the venetoclax arm and 2 (2.9%) in the placebo arm had an MRD assessment with blasts <10^−3^. CR/CRi and an MRD response (blasts <10^−3^) was achieved in 9 patients (6.3%, 95% CI 2.9, 11.6) in the venetoclax arm compared with 1 patient (1.5%, 95% CI 0.0, 7.9) in the placebo arm. At the end of cycle 4, 4 patients (2.8%, 95% CI 0.8, 7.0) in the venetoclax arm had achieved CR/CRi and an MRD response (blasts <10^−3^), compared with zero patients within the placebo arm. When narrowing MRD responses to blasts <10^−4^ (assay sensitivity level), 9 patients (6.3%) in the venetoclax arm and zero patients in the placebo arm met this deep remission criterion. At this threshold, MRD (blasts <10^−4^) and CR/CRi responses were observed in 6 patients (4.2%; 95% CI 1.6, 8.9) in the venetoclax arm and in 3 patients (2.1%, 95% CI 0.4, 6.0) at the end of cycle 4. No patients in the placebo arm achieved these milestones.

**Table 5:** MRD assessments at the 6-month follow-up.

MRD response rates	Venetoclax +LDAC (*n* = 143^a^)	Placebo + LDAC (*n* = 68^a^)	*P* value
***Best MRD value, n (%)***			
<10^−3^	12 (8.4)	2 (2.9)	–
<10^−4^	9 (6.3)	0	–
MRD <10^−3^ and CR/CRi response, *n* (%) [95% CI]	9 (6.3) [2.9, 11.6]	1 (1.5) [0.0, 7.9]	0.118
MRD <10^−4^ and CR/CRi response, *n* (%) [95% CI]	6 (4.2) [1.6, 8.9]	0	0.088
MRD <10^−3^ and CR/CRi response at end of cycle 4, *n* (%) [95% CI]	4 (2.8) [0.8, 7.0]	0	0.173
MRD <10^−4^ and CR/CRi response at end of cycle 4, *n* (%) [95% CI]	3 (2.1) [0.4, 6.0]	0	0.218

*CI* confidence interval, *CR* complete remission, *CRi* complete remission with incomplete hematologic recovery, *LDAC* low-dose cytarabine, *MRD* minimal residual disease.

^a^By the 6-month cut-off, 44 patients in the placebo + LDAC arm and 113 patients in the venetoclax +LDAC arm had an MRD assessment.

**Fig. 2:**
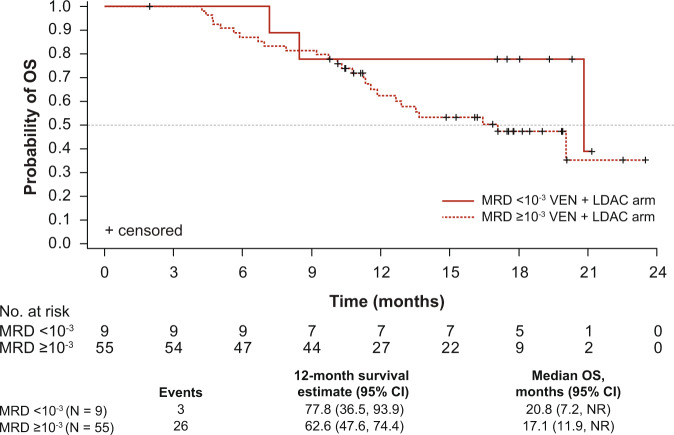
OS in patients treated with venetoclax +LDAC achieving CR/CRi by best post-baseline MRD value (<10^−3^ vs. ≥10^−3^). Kaplan–Meier plot showing the OS rate in patients treated with venetoclax +LDAC who achieved a CR/CRi response, stratified by best post-baseline MRD value (<10^−3^ vs. ≥10^−3^). Unable to graph MRD data for placebo + LDAC arm due to small sample size (data summarized in Table 5). Tick marks indicate censored data. CI confidence interval, CR complete response, CRi CR with incomplete hematologic recovery, LDAC low-dose cytarabine, NR not reached, OS overall survival, VEN, venetoclax, MRD minimal residual disease.

Additionally, as shown in Fig. 2, median OS in the subset of patients achieving CR/CRi in the venetoclax arm is also higher among those who achieved an MRD response (20.8 months, 95% CI 7.2, not reached [NR]) compared with those who did not (17.1 months, 95% CI 11.9, NR). Of note, because of small patient numbers the median OS values for the placebo group could not be calculated.

## Discussion

This 6-month follow-up of the randomized, double-blind, placebo-controlled, multicenter phase 3 VIALE-C study confirms a favorable efficacy and tolerable safety profile of venetoclax in combination with LDAC in patients with AML who are ineligible for intensive chemotherapy due to age or comorbidities.

The primary endpoint of improvement in OS was not met at the time of pre-planned analysis, although imbalances in baseline patient characteristics and early censoring of patients with limited follow-up at the time of the initial data cut-off date may have affected the analysis [12]. With an additional 6-months of follow-up, venetoclax treatment demonstrated significantly increased median OS compared with placebo (8.4 vs. 4.1 months, respectively; HR 0.70, 95% CI 0.50, 0.99, *P* = 0.040). Venetoclax reduced the risk of death by 30% at the 6-month follow-up analysis compared with 25% at the primary analysis.

In addition to this improvement in OS, all remission rates (CR, CR/CRi, and CR/CRh) continued to improve in the venetoclax arm at the 6-month follow-up analysis compared with the primary analysis, with no improvement in rates observed in the placebo arm. Median DoR followed a similar pattern. As of the 6-month follow-up analysis, patients in the venetoclax arm also continued to see improved RBC and platelet transfusion independence and longer EFS compared with the primary analysis (Table 3) with nearly 50% of patients becoming transfusion independent with venetoclax plus LDAC combination therapy. These results demonstrate that venetoclax combined with LDAC is both an effective and durable treatment option for older patients with AML or those with comorbidities who are ineligible for intensive first-line therapies.

Also, of interest, response rates in the subset of patients who had received prior HMA therapy were higher in the venetoclax arm than in the placebo arm. Specifically, within the venetoclax arm, CR, CR/CRi, and CR/CRh were achieved in 7%, 29%, and 21% of patients who had received prior HMA therapy, respectively, compared with 0%, 7%, and 7% within the placebo arm. Similar results were observed in patients who had not received prior HMA therapy with CR, CR/CRi, and CR/CRh responses achieved in 33%, 53%, and 55%, respectively in the venetoclax arm compared with 9%, 15%, and 17%, respectively in the placebo arm (Table 4). Response rates by molecular risk category (P53, NPM1 + , IDH1/2, and FLT3) are of great interest and these data will be the subject of a future dedicated publication. Median OS was also improved for patients in the venetoclax arm compared with the placebo arm, regardless of whether they had (venetoclax vs. placebo, 5.6 vs. 4.1 months), or had not (8.9 vs. 4.7 months) received prior HMA treatment (Fig. 1B).

Additionally, more patients achieved CR/CRi and an MRD response (blasts <10^−3^ and <10^−4^) in the venetoclax arm compared with the placebo arm at the 6-month follow-up (Table 5), and median OS in the subset of patients who achieved CR/CRi was also higher among those who achieved an MRD response (Fig. 2). Although these data did not reach statistical significance, it is likely that the proportion of patients achieving CR/CRi and an MRD response are underestimated, as MRD assessments were not mandated following achievement of a CR/CRi response. Of note, no patients in either arm received allogeneic stem cell transplantation following discontinuation of the study drug.

In addition to improved efficacy, venetoclax maintained a tolerable safety profile when compared with placebo at the 6-month follow-up. Patients in the venetoclax arm experienced similar rates of AEs leading to study drug discontinuation (venetoclax vs. placebo; 26% vs. 24%) and SAEs (67% vs. 62%) compared with patients in the placebo arm. The additional 6-months of follow-up did not lead to an increase in AE frequency compared with that reported in the primary analysis [12]. Similar rates of AEs leading to study drug discontinuation (26% vs. 24%) and SAEs (67% vs. 66%) were reported in the 6-month follow-up and primary analysis, respectively. These data demonstrate that the tolerability of venetoclax is not only comparable to LDAC alone but that there is also no evidence of cumulative toxicity, which is a key consideration for older patients who are at the greatest risk for toxicity [18]. Of note, dose interruptions and/or dose reductions of chemotherapy are standard clinical practices in AML to allow for peripheral blood count recovery in patients with cytopenias who achieve morphological clearance of AML. Therefore, dose interruptions and/or reductions were permitted per study protocol.

Patients in the venetoclax arm also had distinctly lower post-study treatment rates compared with those in the placebo arm at the 6-month follow-up, most notably in the use of intensive chemotherapy (8% vs. 22%, respectively); a numerical increase was noted for patients in the placebo arm compared with the primary analysis (22% vs. 19%, respectively). This is potentially because an increased number of patients in the placebo arm discontinued the study because of disease progression or treatment failure, compared with the venetoclax arm. Inversely, 2- and 4-times as many patients receiving venetoclax achieved CR and CR/CRi, respectively, compared with placebo. These data confirm that more patients in the venetoclax arm derived clinical benefits from the study treatment.

A key secondary endpoint of this study was to evaluate RBC and platelet transfusion independence rates. At the 6-month follow-up, patients in the venetoclax arm had improved transfusion independence rates (RBC, 43.4%; platelet 49.0%; RBC and platelet, 39.2%) compared with patients in the placebo arm (19.1%, 32.4%, and 17.6%, respectively). These rates represent an increase in transfusion independence when compared to the primary analysis (40.6%, 47.6%, and 37.1%, respectively) [12].

The data presented herein are also comparable to those reported in the VIALE-A study (NCT02993523) [19], where venetoclax or placebo was administered in combination with azacitidine to patients with previously untreated AML who were unfit for intensive chemotherapy. This study reported improved median OS and CR rates following venetoclax treatment and the safety profile was also similar to that reported herein, including improved rates of RBC transfusion independence in the azacitidine-venetoclax arm. The consistent improvements in safety and efficacy associated with venetoclax across studies and in combination with different treatments add to the growing body of evidence regarding the safe and predictable effects of venetoclax treatment combinations for older patients with AML.

In conclusion, this 6-month follow-up analysis demonstrates that in addition to its manageable safety profile, venetoclax improves key efficacy measures including OS, CR rates, transfusion independence, and EFS compared with LDAC alone in a durable manner, confirming its promise in this subset of older patients with AML who serve to benefit from an effective, less intensive therapeutic option.

## Supplementary information

Supplementary Table ST1

Reproducibility Checklist

CONSORT Checklist
