# Author Correction: 6-month follow-up of VIALE-C demonstrates improved and durable efficacy in patients with untreated AML ineligible for intensive chemotherapy

**DOI:** 10.1038/s41408-021-00565-6

**Published:** 2021-10-26

**Authors:** Andrew H. Wei, Panayiotis Panayiotidis, Pau Montesinos, Kamel Laribi, Vladimir Ivanov, Inho Kim, Jan Novak, Don A. Stevens, Walter Fiedler, Maria Pagoni, Julie Bergeron, Stephen B. Ting, Jing-Zhou Hou, Achilles Anagnostopoulos, Andrew McDonald, Vidhya Murthy, Takahiro Yamauchi, Jianxiang Wang, Brenda Chyla, Yan Sun, Qi Jiang, Wellington Mendes, John Hayslip, Courtney D. DiNardo

**Affiliations:** 1 The Alfred Hospital and Monash University, Melbourne, VIC Australia; 2 National and Kapodistrian University of Athens Medical School, Laiko General Hospital, Athens, Greece; 3 Hospital Universitario y Politécnico La Fe, Valencia, Spain; 4 CIBERONC, Instituto Carlos III, Madrid, Spain; 5 Centre Hospitalier–Le Mans, Le Mans, France; 6 Almazov National Medical Research Center, Saint Petersburg, Russia; 7 Seoul National University Hospital, Seoul, South Korea; 8 University Hospital Královské Vinohrady and Third Faculty of Medicine, Charles University, Prague, Czech Republic; 9 Norton Cancer Institute, Louisville, KY USA; 10 Hubertus Wald University Cancer Center, University Medical Center Hamburg-Eppendorf, Hamburg, Germany; 11 Evaggelismos General Hospital, Athens, Greece; 12 CIUSSS-EMTL, Installation Maisonneuve-Rosemont, Montreal, QC Canada; 13 Eastern Health and Monash University, Melbourne, VIC Australia; 14 University of Pittsburgh Medical Center (UPMC) Cancer Center, Pittsburgh, PA USA; 15 George Papanicolaou General Hospital, Thessalonica, Greece; 16 Netcare Pretoria East Hospital, Moreletapark, Pretoria, South Africa; 17 Heartlands Hospital, Birmingham, UK; 18 University of Fukui Hospital, Fukui, Japan; 19 Chinese Academy of Medical Sciences, Tianjin, China; 20 AbbVie Inc, North Chicago, IL USA; 21 The University of Texas MD Anderson Cancer Center, Houston, TX USA

**Keywords:** Targeted therapies, Acute myeloid leukaemia

Correction to: *Blood Cancer Journal* 10.1038/s41408-021-00555-8, published online 01 October 2021

The title this article was published with was not correct. It should read “6-month follow-up of VIALE-C demonstrates improved and durable efficacy in patients with untreated AML ineligible for intensive chemotherapy”.

