# Supplementary material for: 6-month follow-up of VIALE-C demonstrates improved and durable efficacy in patients with untreated AML ineligible for intensive chemotherapy
Source: Blood Cancer J. 2021 Oct 1;11(10):163. doi: 10.1038/s41408-021-00555-8 (PMC8486817; doi:10.1038/s41408-021-00555-8)
Supplement: Supplementary file 1 — Supplementary Table ST1 [file 41408_2021_555_MOESM1_ESM.pdf]

Supplementary Material

Supplementary Table S1 Reasons for study drug discontinuation

**Reasons for discontinuation**	**Venetoclax + LDAC (*n* = 143)**	**Placebo + LDAC (*n* = 68)**
**Randomized, *n***	143	68
Treated, *n* (%)	142 (99.3)	68 (100)
Discontinued venetoclax/placebo, *n* (%)	117 (81.8)	63 (92.6)
**Primary reasons for venetoclax/placebo discontinuation, n (%)**		
AE (related to disease progression)	5 (3.5)	3 (4.4)
AE (unrelated to disease progression)	15 (10.5)	6 (8.8)
Patient withdrawal	8 (5.6)	8 (11.8)
Physician decision	8 (5.6)	8 (11.8)
Disease progression[a]	17 (11.9)	12 (17.6)
Morphologic relapse[b]	23 (16.1)	3 (4.4)
Treatment failure[c]	18 (12.6)	13 (19.1)
Death	18 (12.6)	8 (11.8)
Other	5 (3.5)	2 (2.9)

AE adverse event, *CR* complete response, *CRi* CR with incomplete hematologic recovery, *LDAC* low-dose cytarabine.

[a]Disease progression is defined per European LeukemiaNet recommendations: 50% increase in bone marrow or peripheral blasts over baseline or new extramedullary disease. [b]Morphologic relapse is defined as the reappearance of ≥5% blasts after CR/CRi in peripheral blood or bone marrow or the

development of extramedullary disease. [c]Treatment failure is defined as failure to achieve CR, CRi, partial response, or morphologic leukemia-free state, or death from any cause.
